# Memory CD8 T Cells Specific for *Plasmodia* Liver-Stage Antigens Maintain Protracted Protection Against Malaria

**DOI:** 10.3389/fimmu.2012.00370

**Published:** 2012-12-10

**Authors:** Urszula Krzych, Sarat Dalai, Stasya Zarling, Alexander Pichugin

**Affiliations:** ^1^Department of Cellular Immunology, Branch of Military Malaria Vaccine Development, Walter Reed Army Institute of ResearchSilver Spring, MD, USA

**Keywords:** memory T cells, malaria, *Plasmodium*, liver, IL-15, CD8 T cells, mouse model

## Abstract

Immunologic memory induced by pathogenic agents or vaccinations is inextricably linked to long-lasting protection. Adequately maintained memory T and B cell pools assure a fast, effective, and specific response against re-infections. Studies of immune responses amongst residents of malaria endemic areas suggest that memory responses to *Plasmodia* antigens appear to be neither adequately developed nor maintained, because persons who survive episodes of childhood malaria remain vulnerable to persistent or intermittent malaria infections. By contrast, multiple exposures of humans and laboratory rodents to radiation-attenuated *Plasmodia* sporozoites (γ-spz) induces sterile and long-lasting protection against experimental sporozoite challenge. Protection is associated with MHC-class I-dependent CD8 T cells, the key effectors against pre-erythrocytic stage infection. We have adopted the *P. berghei* γ-spz mouse model to study memory CD8 T cells that are specific for antigens expressed by Pb liver-stage (LS) parasites and are found predominantly in the liver. On the basis of phenotypic and functional characteristics, we have demonstrated that liver CD8 T cells form two subsets: CD44^hi^CD62L^lo^KLRG-1^+^CD107^+^CD127^−^CD122^lo^CD8 T effector/effector memory (T_E/EM_) cells that are the dominant IFN-γ producers and CD44^hi^CD62L^hi^KLRG-1^−^CD107^−^CD127^+^CD122^hi^CD8 T central memory (T_CM_) cells. In this review, we discuss our observations concerning the role of CD8 T_E/EM_ and CD8 T_CM_ cells in the maintenance of protracted protective immunity against experimental malaria infection. Finally, we present a hypothesis consistent with a model whereby intrahepatic CD8 T_CM_ cells, that are maintained in part by LS-Ag depot and by IL-15-mediated survival and homeostatic proliferation, form a reservoir of cells ready for conscription to CD8 T_E/EM_ cells needed to prevent re-infections.

## Introduction

One of the cardinal features of Ag-specific immune responses elicited by infections or vaccinations is the persistence of optimally effective memory T cells that are inextricably linked to long-lasting protection (Ahmed and Gray, 1996). Adequately maintained memory T cells assure a fast, effective, and specific response against reoccurring infections. Studies of protective immunity amongst residents of malaria endemic areas indicate that protective immunity to *Plasmodia* antigens develops gradually after multiple exposures over many years and, although associated with a decline in clinical manifestations of the disease, it decays rapidly once exposure to the parasite ceases (Langhorne et al., 2008). However, it is not clear why protection does not persist after malaria infection. We hypothesized (Krzych et al., 2000) that the absence of adequately developed immunologic memory, which stems from the tolerant milieu of the liver (Crispe, 2011), sequestration of the liver-stage antigens (LS-Ags) within hepatocytes, and relatively short duration of the liver-phase infection, is mainly responsible for the lack of lasting protection. Others suggested that a phenomenon known as altered peptide ligand, resulting from polymorphisms at CD8 T cell sites, induces antagonistic effects that interfere with the priming and the survival of memory T cells (Plebanski et al., 1999). Poor immunogenicity may also stem from inadequate immunizing doses or immunologic interferences from blood-stage parasites (Good et al., 2005; Urban et al., 2005). Interestingly, currently conducted studies with the RTS,S vaccine, which is based on *Plasmodium falciparum* circumsporozoite protein (CSP), indicate that protection is conferred to infants and small children but it lasts for a relatively short period of time (Abdulla et al., 2008).

In contrast, exposure of laboratory rodents (Nussenzweig et al., 1967), monkeys (Nussenzweig et al., 1970), and humans (Clyde et al., 1973) to radiation-attenuated (γ) *Plasmodia* sporozoites (γ-spz) induces sterile and long-lasting protection. *Plasmodia* γ-spz-induced protection is multifactorial (Nardin and Nussenzweig, 1993), involving antibody (Egan et al., 1993), CD4 (Nardin et al., 1989), and CD8 T cell (Wizel et al., 1995) responses directed primarily to CSP. However, blood-stage antigens also recall IL-4 producing memory CD4 T cells in protected subjects (Palmer and Krzych, 2002) and LS antigen-1- (LSA-1) specific proliferative T cell responses correlate with protection (Krzych et al., 1995).

CD8 T cells have been considered key effectors against pre-erythrocytic stage infection. Evidence supporting the effector function of CD8 T cells is based on studies in human (Malik et al., 1991) and animal (Schofield et al., 1987; Weiss et al., 1988; Berenzon et al., 2003) models of γ-spz-induced protection, protection induced by genetically attenuated parasites (GAP) (Jobe et al., 2007; Mueller et al., 2007; Tarun et al., 2007; Trimnell et al., 2009) as well as on more recent observations made in models of protection induced by wild type *Plasmodia* sporozoites administered under drug coverage (Nganou-Makamdop et al., 2012). Studies conducted in malaria endemic areas confirm the involvement of effector CD8 T cells in protection (Bejon et al., 2007). The effector function is associated mainly with the production of inflammatory cytokines such as IFN-γ or TNF-α that mediate elimination of the parasite within the hepatocytes by the nitric oxide (NO) pathway (Seguin et al., 1994). CD8 T cells also exhibit cytolytic activity against targets that express antigens belonging to pre-erythrocytic stage parasites (Hill et al., 1991; Malik et al., 1991; Trimnell et al., 2009).

Our lab has adopted the *P. berghei* γ-spz (Pb γ-spz) mouse model to study memory CD8 T cells against experimental malaria infection. CD8 T cells that arise in Pb γ-spz-immunized B6 mice are specific for antigens expressed by the developmentally aborted LS parasites (Berenzon et al., 2003) and antigen experienced CD44^hi^CD8 T cells are found predominantly in the liver (Guebre-Xabier et al., 1999). In this review, we mainly focus on memory CD8 T cells in the maintenance of protracted protection in the Pb γ-spz model. We also comment on studies in other model systems of sterile protection that extend our hypothesis by demonstrating the necessity for late LS-Ags and the induction of effector and memory CD8 T cells for lasting protection (Butler et al., 2011; Teirlinck et al., 2011). We hypothesize that long-term protection to pre-erythrocytic stage infection can be induced and maintained. As such, it requires the formation and persistence of a central memory (T_CM_) CD8 T cell reservoir, which is maintained in part by LS-Ag depot and by IL-15 and which gives rise to IFN-γ producing effector/effector memory (T_E/EM_) CD8 T cells during re-infections.

## *Plasmodia* Parasites in the Mammalian Host; the Importance of the Liver-Stage

*Plasmodia* sporozoites are inoculated into a mammalian host from the salivary glands of an Anopheles mosquito during its blood meal. Sporozoites then quickly travel to the liver via the circulatory and/or lymphatic systems. In the liver, sporozoites undergo further development before they emerge as blood-stage merozoites that infect the red blood cells. Hence, in the mammalian host the *Plasmodium* parasite exhibits three morphologically distinct phases of development: sporozoite-, liver-, and blood-stage. During each stage, the parasite expresses, to some extent, unique protein profiles. The sporozoite-associated proteins facilite parasite invasion of hepatocytes (Rogers et al., 1992; Frevert, 1994; Robson et al., 1995; Fidock et al., 1997) and, under certain conditions, are potent antigens for the induction of cellular and antibody responses. For example, CSP-specific responses correlate with protection in humans (Hoffman et al., 2002) and mice (Schofield et al., 1987). LS-Ags are expressed by the developing parasite within the parasitophorous vacuole in hepatocytes (Mueller et al., 2005; Prudencio et al., 2006) and they are considered to be the major inducers of protective cellular immune responses against the pre-erythrocytic stage parasite (Fidock et al., 1994; Bucci et al., 2000). Proteins that characterize the erythrocytic stage are thought to play a role in the invasion of red blood cells (Holder, 1996).

The mammalian liver plays a key role in the life cycle of the *Plasmodium* parasite as the LS is not only pivotal for survival of the parasite, but it also represents a significant period for the induction, effector phase, and the maintenance of immune memory responses. Understanding immune events that occur in the liver in model systems of protective immunity, as well as during natural infection, will expand our knowledge of organ-specific immune responses to *Plasmodia* antigens and hence facilitate exploitation of these responses to expedite progress in vaccine development against this serious disease.

## A Model of Protracted Protection Induced by *Plasmodia* γ-spz

*Plasmodia* γ-spz-induced sterile and protracted protection is considered the gold standard of anti-malaria vaccines. Like infectious sporozoites, γ-spz carrying CSP and other sporozoite-associated proteins, invade the liver where they undergo aborted development and express LS-Ags (Zechini et al., 1999; Hollingdale and Krzych, 2002). It is believed that antigens expressed by the underdeveloped liver schizonts remain in the liver forming a LS-Ag depot (Scheller and Azad, 1995), which is critical for induction and persistence of Ag-specific protracted protective immunity (Krzych et al., 2000). Treatment of animals with primaquine, a drug which disrupts LS development, concurrently with the γ-spz-immunizations abolishes lasting protection (Scheller and Azad, 1995; Berenzon et al., 2003).

The prevailing state of tolerance in the liver allows for infectious sporozoites to expand and continue their life cycle. However, immunization with γ-spz reverses this tolerance to inflammation, which is needed for the induction as well as persistence of adaptive immune responses (Krzych, 1999). Before the invasion of hepatocytes, Pb γ-spz, like infectious sporozoites, pass through Kupffer cells (KC) (Pradel and Frevert, 2001) changing them to become high IL-12 producers (Steers et al., 2005). In contrast, infectious sporozoites do not activate naïve KC to produce IL-12, and instead downregulate IL-10 (Steers et al., 2005). The importance of IL-12 was demonstrated as an inducer of IFN-γ and iNOS (Sedegah et al., 1994; Seguin et al., 1994) and as a critical cytokine for the development of CD8 T cell responses to pre-erythrocytic stage malaria (Doolan and Hoffman, 1999).

The molecular form of the sporozoites might influence the mode of sporozoite entry into KC, which, in turn, might dictate intracellular localization of sporozoites, as has been recently shown for DC interacting with other parasites (Cervi et al., 2004). On the basis of *in vitro* conducted studies (Pradel and Frevert, 2001; Silvie et al., 2004), the entry of infectious sporozoites is mediated by membrane:membrane fusion and parasites localize in a vacuole that does not co-localize with lysosomes so that sporozoites avoid metabolic degradation before reaching hepatocytes. Conceivably, γ-spz could be internalized by phagocytosis and channeled to phagosomes for metabolic degradation and export by MHC-class II and I molecules. A significant upregulation of MHC-class I is evident on KC after sporozoite challenge of γ-spz-immune mice. In sharp contrast, MHC-class I molecules are downregulated on KC during infection of naïve mice and their APC function is severely reduced (Steers et al., 2005). Inflammatory cytokines increase the expression of MHC-class I-peptide complexes on APC by inducing immune proteosomes for more efficient generation of antigenic peptides for entry into the ER and loading onto empty MHC-class I molecules (Khan et al., 2001). Accordingly, KC from γ-spz-immune/challenged mice present peptides and protein antigens to specific T cells. We propose that a cascade of pro-inflammatory cytokines released during the innate immune response induced by γ-spz leads to temporary local inflammation, which is perceived as a “danger signal” needed to trigger proper responses from the adaptive immune system and lead to long-lasting immune memory (Matzinger, 1994).

## CD8 T Cells Mediate Protective Immunity

Work based on *in vivo* depletion of CD8 T cells (Weiss et al., 1988) unequivocally established CD8 T cells as key effectors in a rodent model of protection against malaria. We confirmed these results by demonstrating a failure to protect β_2_ microglobulin knockout (β_2_m KO) mice (White et al., 1996) and K^b^D^b^ KO mice (Krzych and Schwenk, 2005) immunized with Pb γ-spz. Protection induced by GAP spz is also CD8 T cell dependent as shown by us in β_2_m KO mice immunized with Pb GAP spz (Jobe et al., 2007) and by others in Py GAP protected mice after depletion of CD8 T cells (Trimnell et al., 2009). We also established that effector CD8 T cells are MHC-class I-restricted/dependent because protection is not transferred by γ-spz-immune wt cells into β_2_m KO recipients as CD8 T cells must recognize LS-Ag peptides presented by MHC-class I on APC in the liver. The necessity for cognate peptide recognition by CD8 T_E_ cells was confirmed in experiments using MHC-class I mismatched effector and target hepatocytes (Chakravarty et al., 2007). The need for proximity between effector lymphocytes and target hepatocytes was demonstrated (Rodrigues et al., 1992), as has the ability of hepatocytes to present CSP to CD8 T cells (Bongfen et al., 2007). Target LS-Ags that induce CD8 T cells are being currently defined by us, as well as others (Doolan et al., 2003), using the combination of genomic and proteomic approaches. For example, targeted gene deletion reveals that *Plasmodium* sporozoite low-complexity asparagine-rich protein is essential for early LS development (Aly et al., 2011), whereas FabB/F gene (Vaughan et al., 2009) is essential for late-LS development. Immunization of mice with GAP spz with deletions of each of these genes induces protective immunity. Interestingly, immunization with the late-arresting fabb/f^−^ parasites induces durable protection in several mouse strains and across *Plasmodia* species, presumably because these parasites express a broader repertoire of potential antigens that activate a wider spectra of effector T cells (Butler et al., 2011).

The initial site of induction of liver-resident CD8 T cells remains unclear. It is possible that these cells arise in the liver after interaction with liver APC such as KC or DC that present LS-Ags from developmentally aborted parasites. In support of this hypothesis, we have observed that the number of cCD8α^+^ DC increases in the liver concurrently with the number of Pb γ-spz immunizations, while those in the spleen do not change. Additionally, liver cDCs are very efficient at inducing CD8 T_E/EM_ phenotype and expression of IFN-γ, a process that is both MHC-class I- and IL-12-dependent (Jobe et al., 2009). The possibility of local activation of CD8 T cells by LS-Ags is quite attractive for several reasons, but especially because it enables a rapid response during re-infection. Although it has been shown that activated CD8 T cells home to the liver to be eliminated as a way of purging their destructive effector mechanism (Mehal et al., 1999), there is also evidence that direct activation of naïve CD8 T cells occurs in the liver (Bertolino et al., 2001; Klein and Crispe, 2006). Alternatively, CD8 T cells might be induced in a draining LN by LS-Ag-loaded DCs and during sporozoite challenge migrate to the liver, where they might undergo further expansion. Although quite attractive, migratory DC do not normally express the CD8α^+^ phenotype (Belz et al., 2004) and the cCD8α^+^ DC are the major activators of CD8 T cells in our system (Jobe et al., 2009). Although lack of firm evidence supporting either scenario favors the prevailing view that T cell activation occurs as a result of interaction with DC in the LN, the possibility of an organ-specific activation of CD8 T cells remains very attractive and should be explored further.

The development of TCR transgenic CD8 T cells specific for the *P. yoelii* CSP immunodominant peptide SYVPSAEQI (CS-Tg) has enabled the tracking of early immune responses (Sano et al., 2001). After exposure to bites from Py γ-spz infected mosquitoes, IFN-γ producing CS-Tg T cells were detected in the skin draining LN as early as 48 h after immunization, while spleen and liver responses were not detected until 72 h (Chakravarty et al., 2007). According to these results, CD8 T_E_ cells are generated in the draining LN near the infection (site of mosquito bite) (Chakravarty et al., 2007), and then migrate to liver to kill infected hepatocytes, a process that has been shown to be TAP-dependent, but endosome independent (Cockburn et al., 2011). It is possible that the induction of CD8 T_E_ cells depends on their fine specificity and the site of parasite inoculation. Consequently, CSP-specific CD8 T cells would be induced in the skin draining LN, as sporozoites that are trapped in the skin shed CSP, which could be presented by DCs to T cells. In contrast, LS-Ags, being expressed exclusively in the liver, might activate T cells in the liver, or be transferred either directly or through liver-resident APCs to the liver-draining LNs, to activate T cells there. Owing to the paucity of antigen-specific cells naturally present at the time of immunization or infection, it may not be possible to precisely determine the location of initial priming events.

## Effector Liver CD8 T Cells

Pb γ-spz-immune intrahepatic mononuclear cells (IHMC) contain CD4 and CD8 T cells with inducible CD44^hi^ CD25^hi^ and CD45RB^lo^ phenotypic markers (Guebre-Xabier et al., 1999). Expression of CD45RB, an activation/memory marker, changes from CD45RB^hi^ to CD45RB^lo^ with increased antigen exposure and the state of cellular maturation (Lee et al., 1990; Seder and Ahmed, 2003). Although CD8 CD45RB^lo^ T cells are present in naïve liver, multiple immunizations with γ-spz stabilize and increase the CD45RB^lo^ (or CD62L^lo^) phenotype as well as other phenotypic and functional attributes. For example, enhanced frequencies of CD8 CD45RB^lo^ T cells that secrete IFN-γ and express KLRG-1^hi^CD107^+^ phenotype coincide with the induction of sterile protection (Krzych et al., 2010). These observations are in agreement with the transient expansion of T cells (Ahmed and Gray, 1996; Badovinac et al., 2002) and the role of CD8 T cells as effectors.

Liver CD8 T cells from Pb γ-spz-immunized mice produce enhanced IFN-γ 6 h after challenge and the response peaks around day 7 after challenge (Berenzon et al., 2003). CD8 T cells that produce IFN-γ followed by the induction of nitric oxide synthetase (NOS) (Klotz et al., 1995; Doolan and Hoffman, 1999) might be physiologically relevant to the process of elimination of LS parasites: IFN-γ inhibits the hepatic stages of rodent and human malaria both *in vitro* and *in vivo* (Mellouk et al., 1987); injection of IFN-γ protects mice against sporozoite challenge (Ferreira et al., 1986); and immunization with γ-spz fails to generate protection in IFN-γR KO mice (Tsuji et al., 1995). Moreover, a reduced IFN-γ response from liver CD8 CD44^hi^ T cells correlates with decreased protection in mice (Nganou-Makamdop et al., 2012); and Py CS-Tg T cells eliminate the parasite by a mechanism that depends upon rapid IFN-γ production (Sano et al., 2001).

Secretion of IFN-γ by liver CD8 T cells would preclude the need for direct lysis of hepatocytes, as IFN-γ could suppress parasite growth by the few CD8 T cells that encounter infected hepatocytes. IFN-γ could also contribute to protection indirectly by upregulating MHC-class I and class II molecules and B7-1 and B7-2 co-stimulatory molecules on KC, DC, and hepatocytes. This, in turn, would further promote activation of CD8 T_E_ cells.

The release of IFN-γ, which coincides with the activation of CD8 T cells, is preceded by elevated production of IL-4, which declines when IFN-γ reaches its peak (Krzych et al., 2000). The reciprocal regulation between these two cytokines reflects the precise orchestration of functional activities among T cell subsets induced by γ-spz. It is likely that IL-4 in the liver is produced by NK T cells, whereas IFN-γ is produced primarily by CD8 T cells (Berenzon et al., 2003). This view is in agreement with the observation that CD8 T_E_ cells decline after inflammation has subsided (Badovinac et al., 2002), whereas memory CD8 T cells persist, if they are supported by lymphokine-secreting cells.

In our view, sustained protection requires various CD8 T cell specificities, particularly those belonging to proteins expressed during pre-erythrocytic LS development. It could be envisaged that CSP-specific CD8 T cells initiate the effector stage of protection because they are the first cells to produce IFN-γ upon encountering infectious sporozoites. Protracted protection might require the subsequent activation of a second wave of CD8 T cells specific for epitopes other than CSP, as they would have to target hepatocytes by recognizing LS-Ags. Such concerted and functionally integrated activity provided by CD8 T_E_ cells with multiple specificities might be necessary to provide sustained protection. In their recent study, Butler et al. (2011) propose that GAP parasites arrested during late-LS development induce stronger CD8 T cell responses and durable protection presumably because these parasites contain a richer repertoire of antigens able to induce effector T cells. Similar observations regarding the availability of a more abundant level of late-LS-Ags have been made in a model system of protection induced by *P. bergehi* sporozoites delivered by the intravenous route under a drug coverage (Nganou-Makamdop et al., 2012).

## Memory CD8 T Cells

The formation of optimally effective memory T cells is an essential feature of an adaptive immune response elicited by infections and it is inextricably linked to long-lasting protective immunity (Ahmed and Gray, 1996). Intrahepatic memory CD8 T cells generated by immunization with Pb γ-spz segregate into at least two distinct but developmentally related subsets: the IFN-γ-producing CD44^hi^CD45RB^lo^CD122^lo^CD62L^lo^ phenotype, or T_E/EM_ cells, which can be further subdivided based on the expression of IL7Rα (CD127) and KLRG-1 into T_E_ (CD127^−^KLRG-1^+^) or T_EM_ (CD127^+^KLRG-1^−^); and the indolent IFN-γ producing CD44^hi^CD45RB^hi^CD122^hi^CD62L^lo/hi^ phenotype, hence CD8 T_CM_ cells. The elevated expression of CD122 (IL-15Rα) on CD8 T_CM_ cells suggests that, in contrast to CD8 T_E/EM_ cells, they are IL-15-dependent (Berenzon et al., 2003; Krzych et al., 2010).

Recently, various phenotypic and functional attributes have been evaluated in an effort to understand the differentiation of memory CD8 T cells (Joshi et al., 2011). In addition, asymmetric division (Ciocca et al., 2012), duration and strength of the TCR signal (D’souza and Hedrick, 2006), and inflammatory cytokines (Obar and Lefrancois, 2010) have been examined as requirements for memory T cell development and differentiation. Nonetheless, many questions remain regarding the regulation of memory cell formation and in the case of organ-specific infections, like malaria, additional aspects of memory CD8 T cell development and differentiation need to be considered. We propose that these functionally and phenotypically unique subsets of liver memory CD8 T cells form an interactive network involving different phases of dynamic cell activation and differentiation (Berenzon et al., 2003). The co-presence of distinct subsets within the intrahepatic memory CD8 T cell pool in mice protected against malaria is consistent with an earlier view that virally induced memory CD8 T cells are organized into subsets on the basis of distinct functional activities and the maturation/activation status (Sallusto et al., 1999; Kaech et al., 2002; Wherry et al., 2003).

Similar to the rapid responses mediated by influenza- and Sendai-specific CD8 T_E/EM_ cells (Hogan et al., 2001), intrahepatic CD8 T_E/EM_ cells from Pb γ-spz-immunized mice produce a copious amount of IFN-γ within 1–6 h after infection. Although the pool of CD8 T_E_ cells eventually contracts and the IFN-γ response diminishes, the IFN-γ-producing memory T cells persist in the livers of mice that maintain protracted protection against a re-challenge (Berenzon et al., 2003; Nganou-Makamdop et al., 2012). A decay of protection is typically accompanied by the decline of IFN-γ-producing KLRG-1^hi^CD107^hi^ CD8 T cells (Krzych et al., 2010).

CD8 T_CM_ cells also produce IFN-γ but the responses are low and relatively short-lived. Therefore, these cells do not appear to be directly involved in the elimination of the parasite. Instead, by acquiring the CD122^hi^ phenotype, liver CD8 T_CM_ cells engage in homeostatic proliferation, which qualifies them to function as a reservoir to maintain the size of memory CD8 T cell pools (Judge et al., 2002; Berenzon et al., 2003; Krzych and Schwenk, 2005). The maintenance of memory pools is one of the prerequisites of a memory T cell response because attrition, particularly of CD8 T_E_ cells, is inevitable during any infection (Badovinac et al., 2002).

## Mechanisms for Maintenance of Protection Induced by γ-spz

Evidence from our laboratory indicates that the persistence of memory CD8 T cells correlates with the maintenance of protective immunity. Interestingly, the persistence of memory phenotype CD8 T cells is restricted to liver lymphocytes, because splenic T cells from the same group of γ-spz-immunized mice display a phenotype similar to splenic T cells from naïve mice (Guebre-Xabier et al., 1999).

The persistence of memory CD8 T cells in the liver can be accounted for by several co-existing mechanisms. First, some of the memory T cells detected after immunization may be long-lived memory cells derived from CD8 T_E_ cells that survived the contraction phase after sporozoite challenge and have not lost their replicative abilities (Bannard et al., 2009). Second, the CD44^hi^CD45RB^lo^ CD8 T cells in the livers of long-term immune mice may be derived from cells that constantly ingress to the liver in response to the liver repository of *Plasmodia* antigens. The persisting memory may also be derived from naive CD8 T cells that do not quite acquire effector function during priming and subsequent boost immunizations with Pb γ-spz. It appears unlikely that they traffic to the liver from the spleen, because CD44^hi^ CD8 T cells are not present in the spleens during protracted immunity; it is possible, however, that they traffic from the draining celiac LNs. Irrespective of whether maintenance of protection relies on long-lived intrahepatic memory T cells or T cells that constantly ingress to the liver, both require a repository of *Plasmodia* antigens (Berenzon et al., 2003).

## The LS-Ag Depot is Required for the Maintenance of Protracted Protection

There is ample contradictory evidence with respect to antigen requirement for the persistence of memory T cells (Gray and Matzinger, 1991; Murali-Krishna et al., 1999; Jelley-Gibbs et al., 2005; Zammit et al., 2006). On the basis of results from our laboratory, the persistence of a threshold of accumulated LS-Ags is critical for the maintenance of protective immunity. Administration of primaquine at the time of immunization with Pb γ-spz does not affect protection at primary challenge, but results in a loss of protracted protection, which correlates with a decrease of CD8 T_E/EM_ cells in the liver. The disruption of the intrahepatic-stage parasite development prevents the formation of a local antigen depot, which impedes the conscription of T_CM_ into T_E/EM_ CD8 T cells upon re-challenge. In contrast, delayed administration of primaquine has no effect on lasting protective immunity (Berenzon et al., 2003). These results are indeed expected, as the primary action of primaquine is against LS development, without affecting the sporozoite stage, represented by CSP-specific CD8 T cells (Hafalla et al., 2002; Cockburn et al., 2010).

Although most of the results from viral systems argue against the need for antigen to maintain long-lived memory CD8 T cells (Lau et al., 1994; Murali-Krishna et al., 1999), there is evidence that T cell memory persists if a protracted restimulation of effector T cells is maintained, either by persisting or by cross-reacting environmental antigens (Zinkernagel et al., 1996; Jelley-Gibbs et al., 2005). We suggest that antigen requirements might be quite different in malaria because the parasite exhibits tropism to the liver, which is characterized by immunologic tolerance. The liver antigen repository may be sufficient to play a unique role in distinguishing the “locally” activated liver memory T cells from those found in the spleen or LN.

The precise location of the LS-Ag depot has not been established. In principle, hepatocytes can function as APC. Although there is no evidence that hepatocytes present LS-Ags to activate or to maintain memory T cells, because so few hepatocytes become infected by the invading sporozoites and they also are inefficient as APC (Steers et al., 2005), hepatocytes may provide LS-Ag for cross-presentation by either KC or cDC. It could be envisaged that liver APCs internalize infected hepatocytes and engage in cross-presentation of LS-Ag that gained entry from phagosomes into the MHC-class I pathway. Exogenous particulate Ags were shown to enter the MHC-class I pathway via phagosome-ER fusion (Gagnon et al., 2002) or, as in the case of *Toxoplasma gondii*, fusion of the parasitophorous vacuole with the ER (Goldszmid et al., 2009).

The mechanism of LS-Ag processing and presentation has not been fully investigated; however, we demonstrated that adoptively transferred liver CD11c^+^ DCs isolated from Pb γ-spz-immunized mice confer sterile protection to naive mice during a primary Pb sporozoite challenge (Jobe et al., 2009). In a recent study, it was shown that proliferation of Py CS-Tg CD8 T cells transferred into Py γ-spz-immunized mice can be maintained in the liver for up to 60 days post immunization. Depletion of CD11c prior to immunization abolishes this response, which indicates a role for DC as a possible source of CSP depot (Cockburn et al., 2010).

## Maintenance of Central Memory CD8 T Cells by IL-15

It has been established that IL-15 promotes the survival of long-term memory CD8 T cells by maintaining their homeostatic proliferation, whereas IL-2 stimulates both the initial expansion and subsequent contraction of T lymphocytes (Ku et al., 2000; Li et al., 2001; Waldmann et al., 2001; Sprent and Surh, 2002). Although the CD8 T_CM_ cell subset represents a much smaller fraction of the liver CD8 T cells, twice as many CD8 T_CM_ cells are CD122^hi^ than T_E/EM_ cells, which are primarily CD122^lo^ (Berenzon et al., 2003). On the basis of results from *in vitro* studies, only CD8 T_CM_ cells proliferate in the presence of IL-15 and these cells are severely reduced in IL-15KO mice (Krzych et al., 2009). The enhanced sensitivity of the CD8 T_CM_ cells to reduced levels of IL-15 suggests that this subset preferentially expand upon exposure to elevated levels of IL-15 in the liver. It also implies that an optimal protective response requires a developmental compartmentalization of CD8 T cells, with each subset performing not only a unique role, but also relying on distinct regulatory mechanisms (Krzych et al., 2009).

We explored the issue of IL-15 as a signal required for the maintenance of memory CD8 T cells in IL-15 deficient mice (Krzych et al., 2009). Like wt mice, IL-15 KO mice are protected against a primary challenge administered shortly after the last boost immunization with Pb γ-spz. Protection is short-lived, however, as at re-challenge 2 months later, the IL-15 deficient mice become parasitemic. Analysis of the CD8 T cell subsets at primary challenge show an accumulation of CD8 T_EM_ cells and a very small pool of CD8 T_CM_ cells. It appears, therefore, that in the absence of IL-15, CD8 T_EM_ cells might have developed directly from naïve CD8 T cells as has been shown in other systems (Decaluwe et al., 2010). Without the provision of IL-15, the critical reservoir of memory CD8 T cells is severely reduced and hence unable to sustain a sufficient number of cells needed during re-challenge. We observed a near absence of CD8 T_CM_ cells in IL-15 KO mice prior to secondary challenge. The majority of CD8 T_CM_ cells undergo severe attrition as evidenced by the level of apoptosis mainly within the CD8 T_CM_ cells. Consequently, only ∼1% of the cells remain in IL-15 deficient mice in relation to ∼7% in wt mice. Upon 2° challenge, the majority of CD127^hi^CD8 T_CM_ cells transition to CD127^lo^ phenotype in wt mice, but in IL-15KO mice few cells become CD127^lo^. These observations strongly support our hypothesis that CD8 T_E/EM_ cells are conscripted from the CD8 T_CM_ cells in a continuous, albeit slow, process that occurs in the liver as a result of an increased antigen load after repeated immunizations with γ-spz. An association between sterile protection and increased antigenic load of late-LS parasites has also been demonstrated in another model system using Pb sporozoites (Nganou-Makamdop et al., 2012). The conscription of T_E_ cells from the T_CM_ reservoir also occurs during infection, when large numbers of CD8 T_EM_ cells would be most needed to combat the parasite. For example, it has been shown that a large number of Pb CS_252–260_-specific CD8 T cells is needed to to maintain sterile protection in Balb/c mice challenged with Pb sporozoites (Schmidt et al., 2011). Compared to many bacterial or viral infections, sterile immunity against malaria infection requires 100–1000 fold higher numbers of CD8 T_E_ cells (Schmidt et al., 2008). The requirement for these large numbers of antigen-specific CD8 T cells may be, in part, due to the short time frame of the LS when the parasite is most vulnerable to immune intervention.

In either case, CD8 T_CM_ cell pool is maintained in the liver by IL-15. IL-15 is produced by a variety of cell types (although not by T cells) in response to signaling via TLRs or exposure to type I IFN (Mattei et al., 2001). Pb γ-spz cause upregulation of IL-15 mRNA in KC (Krzych et al., 2009) and liver cDC (Jobe et al., 2007). Upon encounter with specific antigen from the liver repository or upon re-infections, the CD8 T_CM_ cells would be driven to differentiate into the CD44^hi^CD45RB^lo^CD122^lo^ phenotype that is easily triggered by infectious sporozoites to produce IFN-γ. Recently, it was demonstrated that CD8 T cell survival during influenza infection is promoted in the lung by trans-presentation of IL-15 by pulmonary CD8α^+^DCs (McGill et al., 2010). There is evidence (Dubois et al., 2002) that APC retain IL-15 bound to the IL-15Rα chain to transactivate CD8 T cells expressing the IL-15Rβγc complex. On the basis of our previously published results that liver cDC activate CD8 T cells in a MHC-class I-dependent manner to express CD44^hi^, up-regulate IL-15 mRNA (Jobe et al., 2009) and express detectable IL-15 protein (Krzych et al., 2010), together with KC they can function as APCs of LS-Ags and as trans-presenters of IL-15 that target only liver CD8 T_CM_ cells. Our hypothesis is supported by observations from *in vitro* conducted studies that only CD8 T_CM_ cells require trans-presentation of IL-15 in the context of a concurrent signaling via TCR for optimal recall response, as responses by CD8 T_EM_ cells are not augmented by IL-15 (Kokaji et al., 2008).

## Summary

MHC-class I-restricted CD8 T cells have been established as key effectors in protective immunity against pre-erythrocytic-stage malaria infection. The effector function is associated mainly with the production of inflammatory cytokines such as IFN-γ or TNF-α that mediate elimination of the parasite within the hepatocytes by the NO pathway. The success of protection induced by γ-spz depends upon the long-lived intrahepatic memory CD8 T cells that consist of developmentally related subsets as CD8 T_CM_ and CD8 T_EM_ cells. While the CD8 T_EM_ cells are maintained by the antigen-driven conscription of the CD8 T_CM_ cells, the latter representing a very broad spectrum of antigen-specific T cells, is maintained by IL-15 and possibly the LS-Ag depot. This arrangement assures a steady availability of antigen-specific T cells should they be required to combat re-infection. The dependence on specific antigen essentially controls the balance between the two phenotypes and the differential expression of IL-15R prevents the CD8 T_EM_ cells from becoming activated in the event of sporadic co-infections. However, it is the activated status of the intrahepatic memory CD8 T cells that really distinguishes them from the memory CD8 T cells in the spleen and LN as it represents the sentinel of a local, organ-specific infection.

## Conflict of Interest Statement

The authors declare that the research was conducted in the absence of any commercial or financial relationships that could be construed as a potential conflict of interest.
